# The Crucial Question About Contrast-Induced Nephropathy (CIN): Should It Affect Clinical Practice?

**DOI:** 10.3390/ph18040485

**Published:** 2025-03-28

**Authors:** Damian Krzyżak, Marcin Basiak, Adrianna Dec, Marcin Hachuła, Bogusław Okopień

**Affiliations:** Department of Internal Medicine and Clinical Pharmacology, Faculty of Medical Sciences, Medical University of Silesia in Katowice, Medyków 18, 40-752 Katowice, Poland; damiankrzyzak6@gmail.com (D.K.);

**Keywords:** contrast, nephropathy, CIN, CAN

## Abstract

The phenomenon of contrast-induced nephropathy (CIN) and contrast-associated nephropathy (CAN) has been acknowledged for an extensive duration. Recently, there has been a significant rise in research on the topic due to the enhanced availability of imaging investigations. This theory has been thoroughly validated and extensively reported in the scholarly literature. The primary risk factors are chronic kidney disease, diabetes, sepsis, critical illness, circulatory shock, anemia, advanced age, inadequate hydration, and the use of nephrotoxic medications. The principal preventive strategies are the use of iso-osmolar contrast agents and sufficient hydration, which includes the use of intravenous isotonic saline. The administration of N-acetylcysteine has been shown to decrease the occurrence of CIN without impacting outcomes like mortality or the need for dialysis. Recently, a growing number of scholarly studies have contested this phenomenon, or at least, questioned its clinical significance, rendering it primarily a biochemical occurrence. This review aims to evaluate the previously listed studies. Overestimating the possible dangers of post-contrast nephropathy may diminish the sensitivity of imaging tests that may otherwise utilize contrast, so substantially lowering their clinical relevance. This hypothesis is critically significant to science, medicine, and patients, warranting attention despite the necessity for additional research to validate it. The present study demonstrates that the frequency and importance of CIN may be overestimated.

## 1. Introduction

The phenomenon of contrast-induced nephropathy (CIN) and contrast-associated nephropathy (CAN) has been well-documented in the medical literature for several decades. The term CIN was first used in an article published in 1954, in which 20 mL of 50% diodine was used, which stands in significant opposition to the low-osmolar contrasts currently in use [1]. Since then, CIN has become a significant clinical problem [2]. The medical community widely accepts the existence of CIN. However, numerous papers cast doubt on the existence of this phenomenon or, at the very least, force us to deeply consider the cause of contrast-associated nephropathy [3,4,5]. This article provides an overview of the relevant studies and recent research. Other potential factors that may lead to kidney damage include sepsis, critical illness, circulatory shock, dehydration, advanced age, chronic diseases (heart, lung, liver, and kidney), diabetes mellitus, and anemia [6]. This review adopts a narrative approach and does not adhere to any specific criteria for the inclusion or exclusion of scientific articles. Its objective is to provide a general overview of the CIN problem and to identify papers that undermine its existence. Our study attempts to prove that the CIN issue may be somewhat exaggerated.

### 1.1. Definitions and Criteria

Various definitions of CIN exist, including an increase in serum creatinine (SCr) of ≥0.5 mg/dL above baseline at 48 to 72 h after imaging [7,8]. Some studies on post-contrast nephropathy use the definition of acute kidney injury (AKI) as defined by Kidney Disease Improving Global Outcomes (KDIGO) requiring an increase in SCr of at least 0.3 mg/dL (26.5 µmol/L) within 48 h or an increase in SCr to at least 1.5 times the baseline value [8,9]. Adopting a value of 0.3 mg/dl will increase the sensitivity of diagnosing AKI while decreasing the specificity. Conversely, adopting a value of 0.5 mg/dl will result in an enhancement of specificity, albeit at the expense of sensitivity.

A widely accepted criterion in the medical community for determining whether to administer contrast during an imaging study is the patient’s creatinine level. Several papers detail the cut-off points and corresponding risks [7,10]. For example, above 45 mL/min/1.73 m^2^, the risk is assumed to be negligible. Between 30 and 44 mL/min/1.73 m^2^, the risk is moderate unless diabetes is present, which increases the risk of CIN. Below 30 mL/min/1.73 m^2^, the risk is high [10].

### 1.2. Historical Context and Evolution

The history of CIN reflects the evolving understanding of renal physiology and the impact of contrast media. The initial use of high-osmolar contrast agents in the mid-20th century marked the beginning of recognized contrast nephropathy [1]. Over the decades, technological advancements and the introduction of low-osmolar and iso-osmolar contrast agents have significantly mitigated the incidence of CIN [11,12]. The first use of high-osmolar contrast media (HOCM) was in the 1920s at the Mayo Clinic by Osborne and colleagues. Osborne and colleagues originally used this 10% solution of sodium iodide to treat syphilis. The first imaging procedure was the pyelogram. Metrizamide (Amipaque) was the first low-osmolar contrast media (LOCM), developed in 1969 and first used in 1972 [13]. In 1983, researchers published a phase 2 study for the first iso-osmolar contrast media (IOCM) agent [14]. A meta-analysis encompassing 25 studies demonstrated that the utilization of low-osmolal agents in comparison with high-osmolal agents resulted in a 39% reduction in the likelihood of an increase in creatinine levels of 44 umol/L [11]. The division of contrasts according to osmolality and amount of iodine is demonstrated in Table 1. This evolution highlights the importance of continually reassessing medical guidelines in light of new evidence and innovations.

### 1.3. Pathogenesis

The etiology of contrast-induced acute kidney injury (CI-AKI) entails the direct cytotoxic effects of iodine contrast media on renal tubular epithelial cells and vascular endothelial cells, resulting in mitochondrial malfunction, apoptosis, necrosis, and interstitial inflammation. Moreover, indirect effects encompass alterations in renal hemodynamics, leading to intramedullary ischemia and hypoxia, as well as the overproduction of reactive oxygen species (ROS), which detrimentally affect renal function [17,18]. Oxidative stress has been demonstrated to exert a deleterious effect on renal function in a multifactorial manner. In an in vivo mouse model, the decline in mitochondrial transcription factor A (TFAM) and mitochondrial DNA (mtDNA) copy number, which occurs in HK2 cells under conditions of oxidative stress, was found to be reversible following the downregulation of mitochondrial reactive oxygen species (mtROS). Oxidative stress reduction has been shown to decrease TFAM transcription and increase Lon protease-mediated TFAM degradation [19]. In a separate study, reperfusion of ischemic tissues was found to induce the opening of the mitochondrial permeability transition (MPT) pore, depolarization of the mitochondrial membrane, decreased ATP synthesis, and, consequently, increased oxidative stress [20]. In a further study, contrast media-induced mitophagy was found to be abolished when phosphatase and tensin homolog (PTEN)-induced kinase 1 (PINK1) or parkin RBR E3 ubiquitin protein ligase (PARK2) were silenced. PINK1-Parkin-mediated mitophagy plays a protective role in contrast-induced AKI by reducing nucleotide-binding oligomerization domain-like pyrin domain-containing protein 3 (NLRP3) inflammasome activation [21].

### 1.4. Risk Factors

#### 1.4.1. Intrinsic

Previous chronic kidney disease is the most significant risk factor for CI-AKI post-percutaneous coronary intervention (PCI). Patients with Chronic Kidney Disease (CKD) are also at risk of complications such as ischemic heart disease and micro-embolization during angioplasty. Diabetes mellitus significantly increases the risk of CI-AKI, especially when combined with CKD [7,18]. Other factors include advanced age and albuminuria [22].

#### 1.4.2. Extrinsic

It would be beneficial to focus particular attention on nephrotoxic drugs that are known to induce acute renal injury following (computed tomography) CT imaging, even in the absence of contrast medium administration. Such drugs include angiotensin-converting enzyme (ACE) inhibitors, hydrochlorothiazides, and loop diuretics [22]. These factors are associated with hypoperfusion rather than direct kidney damage. Some authors posit that a glomerular filtration rate (GFR) of less than 30 mL/min/m^2^ may serve as a threshold below which the likelihood of contrast-induced nephropathy is elevated [10]. Such a GFR is never observed in isolation; it is frequently accompanied by multimorbidity and the use of the aforementioned drugs, which in itself markedly elevates the incidence of AKI, irrespective of imaging studies or contrast administration. It would be beneficial to determine whether contrast administration is the cause of AKI or whether it is merely a coincidental occurrence without a causal relationship. Additionally, nephrotoxic drugs encompass a range of antibiotics, including high-risk (vancomycin and aminoglycosides) and low risk (ciprofloxacin and ceftriaxone), as well as drugs utilized for diabetes management, such as metformin, and numerous other pharmaceuticals [23]. The primary concern related to metformin pertains to the risk associated with a GFR below 30 mg/dL, which is known to predispose to lactic acidosis [24]. Furthermore, the role of poor hydration as a risk factor should not be underestimated [22]. In the current medical context, it is challenging to identify a patient who requires a contrast-enhanced examination in the acute phase and is not on any nephrotoxic drugs.

Concerns are raised about patients in the Intensive Care Unit (ICU) who need imaging procedures and are also taking nephrotoxic drugs like vancomycin and amphotericin B. This could cause these patients to be wrongly diagnosed with contrast-induced nephropathy (CIN), even though AKI often has different causes [25]. Additionally, cancer patients often necessitate follow-up examinations, including CT scans with contrast, while undergoing nephrotoxic treatment. A study revealed that the administration of contrast within one week before cisplatin (a nephrotoxic drug) administration was associated with a 2.56 increase in the risk of CIN compared with the group in which no contrast was used [25]. This makes it difficult to establish a causal relationship between contrast and AKI, particularly in populations with GFR < 30, due to the difficulty in recruiting a sufficient number of patients and the avoidance of contrast administration in this patient group. Several studies have been conducted to compare the risks associated with intravenous versus intra-arterial contrast administration. The results of these studies are conflicting: on the one hand, they confirm a higher risk with intra-arterial administration [26,27], and on the other, they find no difference between the routes [28]. In addition, it was determined many years ago that the administration of high-osmolar contrast medium was associated with an elevated risk of CIN [29].

### 1.5. Emerging Biomarkers and Diagnostic Tools

Recent studies have concentrated on discovering new biomarkers for the early detection of CIN. Biomarkers including neutrophil gelatinase-associated lipocalin (NGAL) [30], cystatin C [31], and kidney injury molecule-1 (KIM-1) [32] have demonstrated the potential to forecast renal injury more effectively than conventional markers such as serum creatinine. These advancements may facilitate more prompt and precise interventions, which could decrease both the occurrence and intensity of CIN. The primary constraint imposed by these markers pertains to the challenge of determining a cut-off point for, exemplified by NGAL, which is indispensable for the diagnosis of AKI in patients with CKD [33]. In the case of CIN, an increase in creatinine is observed within 12 h [34], whereas NGAL rises after a mere 3 h [35]. Creatinine is a prevalent and relatively inexpensive marker, yet it exhibits a delay in elevation relative to other biomarkers, such as the aforementioned one.

### 1.6. Prevention Strategies

New guidelines published in 2023 by the American College of Radiology recommend using intravenous isotonic saline before and after contrast administration for high-risk patients. As previously stated, dehydration is a significant risk factor for kidney damage. This prevention strategy is therefore essential [22]. Other potential nephroprotective candidates include N-acetylcysteine (NAC) [36,37,38,39,40,41,42,43,44,45] and sodium bicarbonate [44,45,46,47]. One of the earliest prospective studies on the subject demonstrated a statistically significant decrease in creatinine levels after 48 h, while the control group exhibited a statistically insignificant increase [36]. A meta-analysis of the aforementioned studies demonstrated the impact of NAC on the prevalence of CIN; however, it did not reveal any influence on the necessity for kidney replacement therapy, mortality, or persistent renal impairment [48]. In addition, agents such as alprostadil [49,50], misoprostol [51], nifedipine [51], theophylline [51], and vitamin E [40] have also been investigated in single articles in the context of nephroprotection.

N-acetylcysteine amide (NACA) and NAC have been shown to prevent CIN in vitro and in vivo models. The mechanism of action of these compounds involves the inhibition of the p38 MAPK pathway. Additionally, the researchers demonstrated that contrast exposure resulted in thioredoxin-1 (Trx1) downregulation and increased apoptosis signal-regulating kinase 1 (ASK1)/p38 MAPK phosphorylation. The administration of NACA and NAC reversed this process and prevented damage to renal tubular cells via an apoptosis mechanism. Furthermore, NACA has also been shown to reduce oxidative stress [52]. The study, which involved a comparison of different hydration protocols involving 150 milliequivalents of sodium bicarbonate in 1000 mL of 0.9% sodium chloride (NaCl) with those involving 1000 mL of intravenous saline infusion and 150 mg/kg of NAC in 1000 millilitres of 0.9% sodium chloride, revealed no significant differences in the incidence of contrast-induced nephropathy (CIN) among patients with moderate or high risk of CIN. The solutions were administered at a rate of 350 mL/hr for 3 h, and the total volume of non-ionic, low-osmolality contrast agent administered to patients was less than 100 mL [44].

Hydration remains a cornerstone in preventing CIN, with isotonic saline being the most commonly recommended solution. However, the optimal volume, timing, and type of hydration continue to be debated.

Studies on N-acetylcysteine as a protective agent have shown mixed results, indicating the need for further research [18,22]. The PRESERVE trial [53] and the AMACING trial [54] did not find significant differences in major adverse kidney events between patients receiving various prophylactic treatments [18,22]. The utilization of low osmolality agents as opposed to high osmolality agents has been demonstrated to result in a reduction in the risk of post-contrast nephropathy. This risk reduction is particularly pronounced in patients with pre-existing renal failure [11]. The administration of diuretics and ACE inhibitors has been observed to potentially exacerbate CIN through the impairment of renal perfusion. However, adequate hydration has been shown to mitigate this risk, potentially obviating the need for discontinuation of the relevant medications [55].

### 1.7. Advances in Imaging Technology

Advancements in imaging technology, such as low-dose radiation CT and advanced magnetic resonance imaging (MRI) techniques, have significantly reduced the need for contrast agents. The study, which was published in 2024 in the European Heart Journal, reported that these technologies could reduce CIN risk by up to 30% [7,56]. One of the most significant limitations pertains to the cost of the methods and their distribution [57]. The expense of a conventional MRI apparatus is substantial, often surpassing USD 1 million. Consequently, endeavours have been undertaken to substantially reduce this expense to below USD 50,000 [58]. These endeavours include the conceptualization of portable devices, a development that would enhance their accessibility and universality [59].

## 2. Controversies and Counterarguments

### 2.1. GFR Under 30 mL/min/1.73 m^2^

In a study involving 12,508 propensity score-matched patients, a positive correlation between a decrease in GFR and an increase in the incidence of CIN was observed. However, the difference between the group of patients who received contrast and those who did not did not reach statistical significance. Of particular interest is the group with a GFR of less than 30 mL/min/1.73 m^2^, comprising 1486 patients. This group also demonstrated no statistically significant difference between the groups, with an odds ratio of 0.97 (95% CI: 0.72, 1.30), *p* value = 0.89 [4].

Furthermore, data from a propensity score-adjusted retrospective study, which included patients with Chronic Kidney Disease stages III, IV, and V (491 contrast and 491 non-contrast), were analyzed, revealing no correlation between contrast administration and 30-day emergent dialysis, mortality, or AKI [60]. These findings challenge previous assumptions regarding CIN risk stratification based on GFR [10]. Additionally, a study involving a small cohort of patients, including those with GFR subgroups of 15–29 and <15, also did not demonstrate a statistically significant effect of contrast on AKI occurrence or the need for dialysis [61]. However, it is important to note that calculations for GFR groups < 30 were imprecise (i.e., wide 95% CIs) due to the limited sample size. In another study involving 52,411 individuals, no evidence was found to suggest that contrast administration contributed to the development of acute kidney injury, even in cases with GFR below 30 mL/min [62]. This raises the need to reconsider the long-standing belief that a GFR above 30 mL/min is a safe threshold, as such a limit may not be clinically relevant. Moreover, this threshold significantly restricts the number of contrast-enhanced studies that provide greater diagnostic value.

### 2.2. Meta-Analyses and Literature Analysis

A meta-analysis involving 13 non-randomized studies and 25,950 patients demonstrated no difference between the group of patients who received contrast and the control group in terms of the rate of AKI, the need for dialysis, and mortality [5]. In the aforementioned study, similar relationships were observed in patients with diabetes and renal insufficiency. The authors seek to elucidate the comparable prevalence of AKI across groups. Initially, they posit that there is no correlation between the frequency of this disease entity and the administration of contrast media. Conversely, they argue that the design of the included studies, which were observational and non-randomized, may have introduced a degree of selection bias. For instance, clinicians may have been inclined to avoid administering contrast in patients at higher risk of developing this complication.

The article by Ehrmann et al. draws attention to the possibility of bias due to the belief in an increase in the rate of AKI after contrast administration [63]. An analysis of medical databases until 31 December 2015, after consideration of exclusion criteria, found only ten papers evaluating ICU patients for CIN with a control group. Interestingly, some even reported a lower risk of AKI in the study group. Of the papers identified, only four performed statistical risk adjustments for baseline prognostic risk factors of AKI. Three of these employed patient matching as a starting point for the quantitative Bayesian meta-analysis performed by Ehrmann’s teams. The remaining six papers had no statistical risk adjustment. An analysis of these three papers showed no statistically significant difference between the group that received contrast and the control group in the rate of AKI. Also, the a priori relative effective sample size (RESS) value that proves contrast is bad for the kidneys should be at least three to twelve times higher than the value that comes from objective analysis. The authors also indicate that their results do not necessarily contradict studies in animal models. Contrast toxicity may be clinically insignificant in ICU patients due to the numerous renal damage processes already underway.

A meta-analysis of 28 papers covering 107,335 patients revealed no statistically significant difference in the rate of AKI between CECT and non-contrast CT [64]. All the included studies were observational, with the majority being retrospective. Six papers included only ED patients, while seven included ICU patients. Some papers also assessed the need for renal replacement therapy and all-cause mortality at various points, except one paper, which only assessed mortality in hospitals. There was no statistically significant difference between the groups in terms of the need for renal replacement therapy or mortality. The authors reiterate the methodological imperfections of previous studies, including the lack of control groups, which allowed for the assumption that kidney damage caused by another cause was an effect of contrast toxicity and the selection bias towards a sicker patient.

Another large meta-analysis involving 25,950 patients also demonstrated that contrast administration did not affect the risk of death, dialysis, or the occurrence of AKI [5]. This study included only observational studies and did not involve randomized controlled trials. Only two of the thirteen studies analyzed showed an association between contrast and the occurrence of CIN [65,66]. The meta-analysis did not show such an association. The authors observe that a comparable risk of acute kidney injury has been documented in patients with renal insufficiency or diabetes who have undergone the administration of HOCM. Furthermore, the authors inquire as to the potential explanations for the absence of discrepancies in AKI rates between studies with and without contrast. The initial hypothesis for the occurrence of AKI in the contrast group is that it can be precipitated by contrast medium-independent sources. The second is that a significant clinical disparity between groups may exist due to the lack of randomization of groups in the studies.

A separate study, encompassing 32,161 patients in a retrospective analysis of previously published studies, demonstrated a comparable incidence of elevated creatinine levels in patients who did not receive contrast and those who did [67]. The investigation encompassed patients with documented creatinine values over five consecutive days. Over fifty per cent of the patients demonstrated a creatinine elevation of at least 25 per cent, whereas forty per cent of the patients showed an increase of at least 0.4 mg/dL. These alterations transpired in both patients with normal baseline creatinine levels and those with increased levels. This frequency was analogous to earlier published studies regarding the incidence of CIN in patients administered contrast.

One study challenged the link between contrast administration and renal damage as early as 2006 [68]. After analyzing 3081 publications from 1966 to 2004 for keywords, the authors were only able to identify 40 articles that assessed renal function after contrast administration. Only two papers had a matched control group, and both showed no statistically significant difference [65,69]. The research conducted by Heller et al. demonstrated an increased incidence of CIN in the low-osmolality contrast group, which was not observed in the high-osmolality group. The authors hypothesize that this discrepancy may be attributed to selection bias, given that the sicker patients received low-osmolality contrast [69]. The date of publication in both cases emphasizes the fact that the evidence for the lack of a causal relationship between contrast and kidney damage has long existed.

### 2.3. Intensive Care Unit and Emergency Department Patients

A significant proportion of subsequent work was conducted in an emergency department or intensive care unit, possibly due to the more prevalent requirement for contrast-enhanced CT, irrespective of baseline GFR [8,9,63].

A further retrospective study in the emergency department (ED), involving 17,934 patients, including 7201 contrast-enhanced CT scans, found that for patients with creatinine levels below 4 mg/dL, intravenous contrast administration during CT did not increase the risk of AKI or the occurrence of chronic kidney disease, kidney transplantation, or dialysis within six months [8]. In the group with creatinine above 4 mg/dL, there was also no correlation between AKI and contrast-enhanced CT. It is important to note that the study group was relatively small (493 patients and 53 CT scans with contrast) and that the range of creatinine levels was high (4.0 mg/dL to 21.1 mg/dL).

Furthermore, in a study that included ED patients and those meeting Sepsis-2 criteria, there was no difference in the rate of AKI between contrast-enhanced CT (CECT), unenhanced CT, and no CT [9]. No such relationship was observed in a cohort of over 4000 patients, including the subgroup with the lowest baseline renal function. Patients with sepsis represent a distinct population, characterized by an elevated risk of developing AKI, which is further increased in the context of septic shock. The underlying pathophysiology of septic shock is multifactorial. Furthermore, the authors highlighted that the perception of contrast nephrotoxicity has resulted in the avoidance of numerous contrast studies, which are occasionally essential for identifying the source of infection or excluding alternative diagnoses. Furthermore, the authors hypothesized that, based on the presented data, the avoidance of contrast studies is likely unjustified. The principal advantages of this study are the large population size and the propensity score matching analysis. However, the data are from a single centre and the study is retrospective, which represents the main limitations.

A study of 3848 cancer patients admitted to the ICU showed that there were no significant differences between the group that received contrast and the control group [70]. Additionally, the analysis demonstrated an association between an increase in creatinine levels, non-renal SOFA, and male gender. However, eGFR after contrast administration was only associated with baseline eGFR. The authors observed that the elevation in creatinine levels also manifested in the cohort that did not undergo a CT scan. This may indicate the inherent nature of critically ill patients, in whom fluctuations in creatinine levels are observed irrespective of the imaging studies conducted, due to the underlying disease process that precipitates admission to the Intensive Care Unit. A post hoc analysis was also conducted on the two groups, one that had undergone CT with enhancement and the other that had undergone CT without enhancement. No differences were observed regarding dialysis initiation, mean length of stay, or mortality. On the other hand, the data revealed that patients undergoing a CT scan, regardless of the use of a contrast agent, experienced longer hospital stays and a higher death rate. This phenomenon could be attributed to the fact that patients who required a CT scan were more severely ill than those who did not. The above-mentioned analyses suggest that the patient’s condition has a greater influence on the hard endpoints than the potential use of contrast. Furthermore, contrast administration does not act as an independent risk factor for AKI, dialysis, or 30-day mortality, even among patients with compromised renal function or comorbidities [71]. Patients who developed acute kidney injury were more likely to require dialysis and to die, but the authors found no association between contrast administration and these events.

### 2.4. Single-Centre Studies

Several studies challenge the conventional wisdom surrounding CIN. For instance, a study by Zungur et al. [3] questioned the direct causative role of contrast media in renal impairment, suggesting that underlying comorbidities and hemodynamic instability might play more substantial roles. However, this study also demonstrated a positive correlation between the amount of contrast media administered and the incidence of contrast-induced nephropathy (CIN). These findings underscore the need for a nuanced approach when diagnosing and managing CIN, considering the multifactorial nature of kidney injury in patients undergoing contrast-enhanced procedures.

A large, single-centre, retrospective analysis of 53,439 patients, involving 157,140 scans after propensity score adjustment, demonstrated no statistically significant difference in the rate of AKI between contrast and non-contrast groups, regardless of baseline CIN risk [7]. Notably, the above study employed several distinguishing features. The large study group permitted the consideration of multimorbidity and other risk factors that increase the risk of AKI. Furthermore, statistical techniques were employed to level demographic variables. Additionally, a counterfactual analysis was conducted to determine a potential cause-and-effect relationship. A group of patients who underwent both CT scans with and without contrast were analyzed, which was not feasible in retrospective studies accompanied by selection bias.

A prospective study on a group of 716 individuals estimated eGFR and demonstrated comparable independent changes in patients who underwent MRI or CT, regardless of contrast administration [72]. This may correspond to natural fluctuations. Of particular interest is that CIN (defined as an increase in more than 0.5 mg/dL) developed only in the control groups (2 MRI, 1 CT) without statistical significance. The incidence of an increase of 25% was also higher in the control groups. It is notable that the above study revealed that neither age nor history of kidney disease had any effect on the variability in glomerular filtration rates. It should be indicated that the control group was smaller than the study group.

A study comparing contrast-enhanced CT and gadolinium-based MR showed no statistically significant differences between these diagnostic tests in the rate of renal damage, except the subgroup of patients with systolic blood pressure below 110 mm Hg [73]. After matching the data, the significance was lost, possibly due to the smaller number of patients.

It is noteworthy that in one study, GFR even improved after contrast administration (−3.06 ± 8.39, 112 patients, GFR 30–60 mL/min/1.73 m^2^) [74]. This may be attributed to the preparation of the patients for the study, which involved intravenous fluid supply, a nephroprotective factor, particularly isotonic fluids [75]. Oral hydration is as effective as intravenous hydration in Chronic Kidney Disease [76].

Even if we accept the assumption that CIN exists, we believe that its clinical relevance should be considered. In a study on a population of patients with acute ischemic stroke, 20 of 189 developed CIN, while none required dialysis [77]. This raises the question of whether the increase in creatinine observed in AKI has clinical implications or whether it remains merely a laboratory parameter that fluctuates over time. In our opinion, this requires more prospective studies.

### 2.5. Multicentre

In the CONNECT study, the authors demonstrated that only pre-existing kidney impairment is a risk factor for CIN [78]. No such association was shown for nephrotoxic drugs, diabetes, or heart failure. The population consisted of 493 patients. A study evaluating the excretion of kidney injury molecule-1 (KIM-1) and neutrophil gelatinase-associated lipocalin (N-GAL) demonstrated that contrast administration did not result in tubular injury in either the CIN or control groups [79].

### 2.6. Summary

In conclusion, the above studies demonstrate that there is no association between contrast administration and the occurrence of AKI, regardless of the study design (single-centre vs. multicentre, prospective vs. retrospective, and propensity score-matched patients vs. non-matched). The reader will find a summary of the studies included in Section 3, along with a discussion of their methodological limitations, in Table 2. The issue of multimorbidity and hemodynamic abnormalities was brought to the fore. A comparison of groups of patients who received contrast prior to diagnostic testing with those who did not receive it repeatedly failed to achieve statistical significance in various papers. This encompasses patients exhibiting a glomerular filtration rate (GFR) of less than 30 mL/min/m^2^, as well as those with a GFR of less than 15 mL/min/m^2^. However, due to the current recommendations to avoid contrast-enhanced studies in this group of patients, the study populations were considerably smaller than those with GFR > 30 mL/min/m^2^. Furthermore, studies have demonstrated that the mere occurrence of AKI does not inevitably lead to adverse outcomes such as the necessity for dialysis, kidney transplantation, or increased mortality in patients with AKI. These relationships have also been observed in AKI risk groups, including patients with diabetes or renal insufficiency. Some of the aforementioned analyses were conducted on groups of patients in the most severe condition, including those in the ICU. For patients meeting the criteria for a diagnosis of sepsis, the situation was similar. Furthermore, it was observed that the elevation in creatinine levels manifested in the cohort that did not undergo imaging studies, which can be attributed to the intrinsic characteristics of critically ill patients. Additionally, no evidence of tubular damage was identified in the context of CIN. In the circumstances of acute kidney injury, it is of the utmost importance to take into account the patient’s overall state of health. Numerous factors, including other diseases like diabetes and chronic kidney disease, and the use of certain drugs with nephrotoxic effects, can cause AKI. The previously cited studies question the existence and significance of CIN. Therefore, it can be argued that the issue of CIN is probably overexposed, which surely needs to be proven by further studies.

## 3. Future Directions

In light of the ongoing debates and emerging evidence, future research should prioritize a deeper understanding of the mechanisms underlying CIN. This should include an investigation into the existence of CIN and its clinical significance, the identification of high-risk populations, and the refinement of prevention strategies. The integration of personalized medicine approaches and continued advances in imaging technologies has the potential to minimize the risk of CIN while maximizing diagnostic efficacy [18,22]. Notwithstanding the numerous arguments cited above that cast doubt on the unequivocal occurrence of contrast-induced nephropathy (CIN), it would be imprudent for the scientific community to draw hasty conclusions in the absence of certainty, in line with the precautionary principle that applies to vulnerable populations. Patients requiring contrast-enhanced imaging studies often belong to populations at particular risk of developing nephropathy (e.g., elderly people with multimorbidity taking multiple nephrotoxic drugs, ICU patients, etc.). Furthermore, as demonstrated in this article, the identification of suitable markers for AKI remains a continuous endeavour, and there is currently no consensus on its definition. The modification of guidelines necessitates a careful and well-founded approach to this matter The objective of the current review was to identify studies that call into question the existence and relevance of CIN. As authors, we are of the opinion that CIN may not exist in the currently accepted sense.

## 4. Conclusions

The concept of CIN remains contentious, with evidence both supporting and questioning its clinical significance. These considerations prompt us to reflect on the theme of CIN. Does it exist? If it does, does it have clinical implications? If so, are they significant enough to limit diagnostic imaging with contrast, for example, because of increased creatinine levels. The question then becomes whether the risk of not detecting a lesion due to a lack of contrast outweighs the risk and clinical relevance of the problem discussed above. It is these authors’ opinion, based on the evidence available, that the number of tests with contrast should be increased, given the questionable existence of CIN. While preventive measures are crucial, the decision to use contrast agents should balance the diagnostic benefits against potential risks, particularly in high-risk patients suffering from multimorbidity or those prescribed nephrotoxic medications. These groups are especially vulnerable to developing CIN. As the field evolves, ongoing research and technological advancements will likely provide clearer guidelines for managing and preventing CIN. The comprehensive review of CIN and CAN highlights the complexity and evolving nature of this medical phenomenon. Although we have made significant progress in understanding and mitigating the risks associated with contrast media, further research and innovation are needed to refine preventive strategies and improve patient outcomes. Examples include new contrast agents, substances to prevent CIN, or better hydration protocols. A balanced, evidence-based approach that considers individual patient risks and emerging technologies will be key to advancing the field and improving clinical practice. A notable strength of this narrative review lies in its emphasis on the evidence that calls into question the existence or clinical relevance of CIN. It is hoped that readers inspired by the above review will decide to explore the above topic and conduct prospective randomized studies to expand the knowledge of CIN. 

## Figures and Tables

**Table 1:** Different osmolality contrast media comparison.

Type of Contrast	Contrast Media	Osmolarity mosm/kg H_2_O	Iodine Concentration (mg/mL)
high osmolarity	Meglumine diatrizoate [15]	2070	370
low osmolarity	Iohexol [15]	880	350
low osmolarity	Iopamidol [15]	616	300
iso-osmolar	Iodixanol-320 [16]	290	320

**Table 2:** Articles from Chapter 2 listed in chronological order as published.

Ordinal Number	Year of Publication	Author	Study Limitations	Main Outcome	Patients Population
1	1985	Cramer et al. [65]	Small sample size	Previous uncontrolled studies may have overestimated the risk of renal failure induced by contrast material.	193 enhanced CT 233 control
2	1991	Heller et al. [69]	Small sample size	There does not appear to be a risk of renal impairment from the use of high osmolality radiocontrast material.	292 HOCM 187 LOCM 405 no contrast
3	2002	Mueller et al. [75]	Group could be bigger	Contrast media-associated nephropathy was significantly reduced with isotonic (0.7%, 95% confidence interval, 0.1–1.4%) vs. half-isotonic (2.0%, 95% confidence interval, 1.0–3.1%) hydration (*p* = 0.04).	809 isotonic hydration 811 half-isotonic hydration
4	2005	Polena et al. [66]	Very small sample size	Data demonstrate that iodine contrast media for CT in ICU patients without pre-existing kidney disease can precipitate measurable nephropathic changes.	No data available
5	2006	Rao et al. [68]	Review	Properly controlled clinical studies of intravenously administered radiographic contrast media fail to demonstrate renal damage.	3081 publications
6	2006	Dussol et al. [76]	Small sample size	There was no significant difference between the rate of renal failure in the different arms of the study.	312 patients divided into 4 arms of different hydration protocol
7	2008	Newhouse et al. [67]	Retrospective study	The creatinine level increases in patients who are not receiving contrast material as often as it does. in published series of patients who are receiving contrast material.	32,161 patients who had not received contrast material during the previous 10 days
8	2010	Ng et al. [70]	Retrospective study	Administration of IV contrast medium in oncologic ICU patients with relatively normal creatinine is associated with an increase in creatinine but not beyond that of simply undergoing CT or of a matched non-CT group in ICU.	3848 ICU oncology patients
9	2010	Lencioni et al. [78]	Observational study, with a very small sample size	Use of the iso-osmolar agent iodixanol appears to be associated with a low incidence of CIN in at-risk patients.	493 patients—multicenter
10	2013	McDonald et al. [5]	Review of nonrandomized studies	Controlled contrast medium-induced nephropathy studies demonstrate a similar incidence of AKI, dialysis, and death between the contrast medium group and control group.	13 studies 25,950 patients
11	2013	McDonald et al. [7]	Retrospective study	Following adjustment for presumed risk factors, the incidence of CIN was not significantly different from contrast material-independent AKI.	157,140 scans 53,439 unique patients
12	2014	McDonald et al. [4]	Retrospective study	The risk of AKI is independent of contrast material exposure, even in patients with eGFR of less than 30 mL/min/1.73 m².	12,508 patients 1486 patients with GFR < 30 mL/min/1.73 m^2^
13	2014	Mcdonald et al. [71]	Retrospective study	Intravenous contrast material administration was not associated with excess risk of AKI acute kidney injury, dialysis, or death, even among patients with comorbidities reported to predispose them to nephrotoxicity.	The 1:1 matching based on the propensity score yielded a cohort of 21,346 patients
14	2014	Azzouz et al. [72]	Small sample size	eGFR in outpatients undergoing MRI or CT did vary independently of whether the patient received contrast or not.	716 patients
15	2015	McDonald et al. [60]	Retrospective study	Intravenous contrast material administration was not associated with an increased risk of AKI, emergent dialysis, and short-term mortality in a cohort of patients with diminished renal function.	6902 patients (4496 CKD stage III, matched: 1220 contrast and 1220 noncontrast; 2086 CKD stage IV-V, matched: 491 contrast and 491 noncontrast
16	2015	Garfinkle et al. [61]	Retrospective study, small sample size especially with GFR under 30 mL/min/1.73 m^2^	Incidences of acute kidney injury and dialysis after acute kidney injury attributable to contrast-enhanced CT were statistically insignificant across glomerular filtration rate (GFR) subgroups.	2277 patients 47 patients GFR 15–29 21 patients GFR < 15
17	2015	Koiman et al. [79]	Small sample size	KIM-1 and N-GAL excretion were unaffected by CE-CT both in patients with and without CI-AKI, suggesting that CI-AKI was not accompanied by tubular injury.	511 patients
18	2016	Zungur et al. [3]	Very small sample size	The Mehran risk score (MS) is a predictor of CIN development after transcatheter aortic valve implantation (TAVI). The use of MS in clinical practice may decrease renal complications after TAVI.	93 patients who underwent TAVI
20	2017	Hinson et al. [8]	Retrospective study	In the largest well-controlled study of acute kidney injury following contrast administration in the ED to date (2017), intravenous contrast was not associated with an increased frequency of acute kidney injury.	16,801 patients Emergency Department
21	2018	Aycock et al. [64]	Meta-analysis of observational studies, risk of selection bias	Meta-analysis demonstrated that, compared with non-contrast CT, contrast-enhanced CT was not significantly associated with either acute kidney injury, need for renal replacement therapy, or all-cause mortality.	In total, 28 studies involving 107,335 participants were included in the final analysis, all of which were observational.
22	2019	Puchol et al. [62]	Retrospective study	The administration of intravenous contrast material in the patients studied is not associated with increased acute kidney injury.	52,411 patients Emergency Department
23	2019	Hinson et al. [9]	Retrospective study	Contrast media administration was not associated with an increased incidence of AKI.	4171 patients who met sepsis diagnostic criteria
24	2019	Gorelik et al. [73]	Retrospective and nonrandomized	With the current precautions undertaken, the real-life risk of postcontrast acute kidney injury (PC-AKI) among inpatients undergoing CT is insignificant.	742 magnetic resonance imaging (MRI) 8089 iodine-based contrast agents (IBCA)
25	2020	Rudnick et al. [10]	Review, lack of methodology	In all patients at increased risk for CIN, the risk for CIN needs to be balanced by the risk of not performing an intravenous contrast-enhanced study.	No meta-analysis
26	2020	Delgado et al. [77]	Very small sample size	CIN is relatively common after endovascular treatment, whereas it does not increase the risk of renal replacement therapy.	189 patients underwent endovascular treatment for acute ischemic stroke.
27	2021	López et al. [74]	Very small sample size	The incidence of contrast-induced nephropathy in patients with chronic renal failure and GFR 30mL-60mL/min/1.73 m^2^ is low. The biomarkers of renal function analyzed improve in patients who receive premedication and the minimum dose of contrast material.	112 patients with chronic renal failure

## Data Availability

Not applicable.
